# Changing Patterns of lung, liver, and head and neck non-AIDS-defining cancers relative to HIV status in Tanzania between 2002-2014

**DOI:** 10.1186/s13027-016-0106-5

**Published:** 2016-11-21

**Authors:** Julee A. Campbell, Amr S. Soliman, Crispin Kahesa, Sioban D. Harlow, Diwani Msemo

**Affiliations:** 1 Department of Epidemiology, University of Michigan School of Public Health, 1415 Washington Heights, Ann Arbor, MI 48109 USA; 2 Department of Epidemiology, University of 984395 Nebraska Medical Center College of Public Health, Omaha, NE 68198-4395 USA; 3 Ocean Road Cancer Institute, Ocean Road, Dar es Salaam, Tanzania

**Keywords:** Non-AIDS-defining cancer, HIV, Tanzania, Ocean Road Cancer Institute

## Abstract

**Background:**

Tanzania, like other low-income countries, has an increasing cancer burden that remains underestimated. Infection-associated malignancies, particularly HIV-infection, represent a great proportion of cancer burden in Tanzania and throughout Africa. Availability of HIV treatment and improved survival of HIV patients are suggested factors related to increasing prevalence of non-AIDS-defining cancers (NADCs). This study examined patterns of NADCs and proportions of HIV-positivity at the Ocean Road Cancer Institute (ORCI).

**Methods:**

We reviewed logbooks of all ORCI patients diagnosed and/or treated for lung, liver, and head and neck cancers during 2002–2014. The number of total cancers diagnosed at ORCI during this period was used to calculate proportions of NADCs. We abstracted medical records to obtain demographic and clinical profiles and HIV status information for 1127 patients diagnosed or treated during 2010–2014. Trends in numbers and proportions of NADCs were analyzed using Joinpoint regression. Characteristics of NADC patients were analyzed using multinomial logistic regression.

**Results:**

NADCs diagnosed at ORCI increased by 33.8% from 2002 to 2014 while the proportion of NADCs relative to all cancers significantly decreased from 6.8% in 2002 to 5.6% in 2014 (APC = -2.74%). Numbers and proportions of lung and liver cancers increased compared to all cancer diagnoses from 2002 to 2014. The number of head and neck cancers increased while decreasing proportionally compared to all cancer diagnoses from 2002 to 2014. Among patients with pathologically confirmed NADCs between 2010 and 2014, HIV prevalence showed a non-statistically significant decrease from 8.1 to 7.1% (APC = -3.77%).

**Conclusions:**

Absolute numbers of lung, liver, and head and neck cancers increased at ORCI by 1/3 since 2002. Improving survivorship of HIV patients and varying immunodeficiency status may have contributed to the increasing number of NADCs. Total cancer diagnoses nearly doubled during this period, leading to a smaller relative proportion of NADCs diagnosed in 2014 compared to 2002. Late- stage diagnosis and short survival of NADCs included this study may explain possible underestimation and smaller increase in proportion of these particular NADCs compared to other NADCs studied in Tanzania. The slight decrease in proportion of HIV-positive NADC patients during 2010–2014 may suggest increasing patient longevity and more effective HIV management in Tanzania.

## Background

In 2013, 14.9 million incident cancer cases and 8.2 million deaths were estimated to occur worldwide. Rising prevalence over the past decades lends evidence to cancer as a serious global public health issue [1]. Based on the most recent *GLOBOCAN* report on worldwide cancer incidence and mortality, projections estimate an increase to 19.3 million incident cancer cases annually by 2025 [2]. Of this substantial global disease burden, over half of incident cancer cases and deaths occur in less developed regions and these figures are similarly projected to increase in the next decade [2].

The burden of cancer in Africa is particularly detrimental due to exposure to major risk factors, limited awareness of early symptoms, and limited diagnostic facilities and access to care necessary to detect and treat cancer patients [1, 3]. In Africa, 80% of cancers are first diagnosed at advanced stages due, in part, to lack of adequate screening and early detection [4]. Cancers associated with infectious agents are among the most commonly diagnosed cancers in Africa [3]. Specifically, HIV/AIDS infection and associated immunosuppression has been shown to increase risk for AIDS-defining cancers (ADCs), including Kaposi sarcoma, non-Hodgkin lymphoma, and invasive cervical cancer [5, 6]. ADCs are cancers occurring in HIV-positive individuals, at which point a patient is diagnosed with stage 3 HIV indicating progression to AIDS [7, 8].

As Sub-Saharan Africa accounts for 71% of the 24.7 million global HIV/AIDS cases, infection-associated diseases like cancer are of increasing concern [9]. Comparable with the 4.7% adult HIV prevalence in all of Sub-Saharan Africa, Tanzania had a 5% HIV prevalence in individuals aged 15–49 and 1.4 million people living with HIV in 2013 [9, 10]. Compared with neighboring Kenya (6% adult prevalence) and Zambia (12.5% adult prevalence), Tanzania has seen success in infection control efforts. In the last decade, Tanzania has improved HIV management efforts by increasing access to antiretroviral therapy (ART), with programs implemented nationally in 2004 [9–12]. The number of people living with HIV in Tanzania has declined from 7% in 2003/4 to 5.1% in 2011/12 [9].

As a result of scaling up HIV prevention services and antiretroviral therapy (ART) coverage, studies reveal sustained viral suppression and decreasing burden of ADCs while predicting subsequent increasing burden of non-AIDS-defining cancers (NADCs) in individuals receiving ART [11, 13, 14]. HIV patients treated with ART are found to be at increased risk for non-AIDS health complications, many of which are also associated with increasing age [13]. Recent data suggests increased incidence of liver disease, cardiovascular disease, and cancer in HIV-infected and treated patients compared to age-matched non-HIV infected individuals, further suggesting an association between HIV and cancer [13]. Mechanisms for the association between HIV infection and NADC diagnoses include persistently elevated inflammation during long-term ART treatment [13, 15], low CD4 counts in those on treatment [13, 16], and increased longevity and age [11]. Analysis of aggregate AIDS and cancer registry data in US adults suggests statistically significant excesses in ADCs (KS, NHL, invasive cervical cancer) as well as NADCs potentially influenced by immunosuppression [17]. HIV treatment is reducing direct HIV and AIDS related morbidity and mortality in Tanzania, and research is needed to detect how treatment is changing the profile of diseases resulting from sustained ART such as non-AIDS-defining cancers.

As Tanzania does not currently have a population-based cancer registry, this study was conducted to gain further understanding of Tanzania’s changing cancer burden. This project, undertaken at the ORCI, the main cancer treatment center of Tanzania in Dar es Salaam, built upon our previous work on ano-rectal, squamous cell carcinoma of the eye, and Hodgkin lymphoma NADCs [11]. The first objective of this study was to estimate the temporal pattern for additional types of NADCs (lung, liver, and head and neck) between 2002 and 2014. This time period captured trends in NADCs both before and after ART implementation. Secondly, this study examined proportions of HIV-positive patients diagnosed with NADCs from 2010 to 2014 in order to investigate potential connection between prevalence of HIV-related immunodeficiency and diagnosis of lung, liver, and head and neck NADCs.

## Methods

This retrospective study focused on lung, liver, and head and neck NADCs and was conducted at the Ocean Road Cancer Institute (ORCI) in Dar es Salaam, Tanzania. As the main center for cancer treatment in the country, ORCI receives patients from all geographical regions of Tanzania. An electronic registry at ORCI is still being developed and this study utilized ORCI’s hospital-based cancer registry records [18]. Previous reports on this population characterized ano-rectal, squamous cell carcinoma of the eye, and Hodgkin lymphoma NADC patients [11]. This study subsequently focused on lung, liver, and head and neck NADCs.

Annual logbooks from 2002 to 2014 were reviewed for total number of lung, liver, and head and neck NADC cases diagnosed at ORCI. Data was not collected from 2007, as logbook records from this year were not available. Total numbers of lung, liver, and head and neck cancers as well as all cancers diagnosed per year were collected to calculate annual proportions of each of the 3 NADC types identified during this 12-year period.

Corresponding to annual logbooks, medical records of 1127 patients with lung, liver, head and neck cancers diagnosed from 2010 to 2014 were identified and abstracted. This shorter 5-year retrospective period was chosen to characterize recent NADC trends and patient characteristics in relation to HIV. Variables abstracted from ORCI medical records included sex, age, region of residence, tobacco and alcohol use, history of comorbidities, HIV status, cancer type, cancer stage, date of cancer diagnosis, cancer treatment, cancer recurrence, subsequent cancer diagnoses, and recorded death/mortality. Within medical records, pathology reports were utilized to abstract the site and stage of cancer while radiology and chemotherapy department documents provided information on clinical diagnosis and treatment modality.

Lung cancer diagnosis included patients with both small cell and non-small cell cancer, as pathological diagnoses and did not consistently distinguish between types. Liver cancer diagnoses included patients with a recorded diagnosis of either “hepatocellular carcinoma” or “hepatoma.” Head and neck cancers included cancer sites in the following sites: oropharynx, parotid, tongue, oral cavity, palate, lip, salivary gland, gingiva, intra-oral jaw/mandible/maxilla, tonsil, maxillary sinus, sinonasal, nasal, nasopharynx, larynx, hypopharynx, supra-/epi-glottis, pharynx, neck (non-skin), and sub-mandible and excluded malignancies of the skin, lymph nodes and brain. In this study, head and neck cancers were grouped into sub-categories of “oral cavity/oropharynx,” “nasal cavity/paranasal sinuses,” and “hypopharynx/larynx/trachea” for comparison according to anatomical grouping of head and neck malignancies, described in the World Health Organization International Agency for Research on Cancer (IARC) Classification of Tumors [19].

Patients with the diagnosis of lung, liver, and head and neck cancers identified from the logbooks but not confirmed by medical record pathology reports were not included in this study. For individuals lacking site-specific diagnoses in logbooks, medical records were retrieved, reviewed, and patients were included or excluded based on pathology report criteria. Geographic locations of residence reported in ORCI documents were classified on a regional level to protect patient anonymity for those rare cancers. Discrepancies between demographics reported by logbooks and medical records were resolved by prioritizing data that was listed on the majority of medical record reports. Numbers of recorded tobacco and alcohol users at the time of cancer diagnosis were minimal in this population, as displayed in Table 1, and were combined with individuals with a history of substance use for the comparison of “ever use” to “never use” of tobacco and alcohol between the NADC patient populations.

**Table 1:** Total NADC (lung, liver, and head and neck) patient characteristics, 2010-2014^a^

	All NADCs (*N* = 1127) N (%)	Lung (*N* = 101) N (%)	Liver (*N* = 151) N (%)	Oral cavity/Oropharynx^b^ (*N* = 421) N (%)	Nasal cavity/paranasal sinuses^c^ (*N* = 230) N (%)	Hypopharynx/larynx/trachea^d^ (*N* = 224) N (%)
Female	410 (36.4%)	51 (50.5%)	63 (41.7%)	159 (37.8%)	98 (42.6%)	39 (17.4%)
Mean Age ± SD	54.3 ± 15.8	58.6 ± 13.8	47.1 ± 15.6	56.6 ± 15.2	47.9 ± 16.6	59.3 ± 13.4
Residence in Dar es Salaam	448 (39.8%)	53 (52.5%)	70 (46.4%)	140 (33.3%)	91 (39.6%)	95 (42.4%)
Smoking Status						
Previous	404 (35.9%)	33 (32.7%)	27 (17.9%)	170 (40.4%)	52 (22.6%)	122 (54.5%)
Current	25 (2.2%)	2 (2.0%)	2 (1.3%)	12 (2.9%)	1 (0.4%)	8 (3.6%)
Never	477 (42.3%)	39 (38.6%)	71 (47.0%)	169 (40.1%)	125 (54.4%)	73 (32.6%)
Alcohol use						
Previous	411 (36.5%)	30 (29.7%)	39 (25.8%)	182 (43.2%)	52 (22.6%)	108 (48.2%)
Current	54 (4.8%)	5 (5.0%)	5 (3.3%)	19 (4.5%)	11 (4.8%)	14 (6.3%)
Never	399 (35.4%)	31 (30.7%)	59 (39.1%)	143 (34.0%)	107 (46.5%)	59 (26.3%)
Tuberculosis	94 (8.3%)	26 (25.7%)	4 (8.0%)	32 (22.4%)	17 (22.4%)	15 (19.7%)
Co-morbidities^f^						
Mean count ± SD	0.46 ± 0.76	0.97 ± 0.98	0.77 + 0.93	0.37 ± 0.65	0.34 ± 0.67	0.34 ± 0.63
HIV-status						
Positive	81 (7.2%)	4 (4.0%)	11 (7.3%)	36 (8.6%)	22 (9.6%)	8 (3.6%)
Negative	189 (16.8%)	26 (25.7%)	24 (16.0%)	87 (20.7%)	38 (16.5%)	14 (6.3%)
Unknown	857 (76.0%)	71 (70.3%)	116 (76.8%)	298 (70.8%)	170 (73.9%)	202 (90.2%)
Advanced cancer stage^e^	655 (58.1%)	42 (41.6%)	49 (32.5%)	291 (98.0%)	135 (95.1%)	138 (85.7%)
Cancer Treatment Modality						
Radiotherapy	770 (68.3%)	32 (31.7%)	40 (29.0%)	332 (81.6%)	174 (80.9%)	192 (88.1%)
Chemotherapy	574 (50.9%)	60 (59.4%)	87 (63.0%)	184 (45.3%)	128 (59.8%)	115 (52.5%)
Both	414 (38.6%)	15 (14.9%)	25 (18.1%)	158 (38.7%)	112 (52.1%)	104 (47.3%)
Recurrence	41 (3.6%)	0	0	20 (4.8%)	18 (7.8%)	3 (1.3%)
Subsequent cancer diagnosis (at least one)	159 (14.1%)	34 (33.7%)	30 (19.9%)	37 (8.8%)	39 (17.0%)	19 (8.5%)
Recorded Death	149 (13.2%)	14 (13.9%)	33 (21.9%)	51 (12.1%)	27 (11.7%)	24 (10.7%)

^a^Totals vary due to missing data

^b^Oral cavity/oropharynx cancers include oropharynx, parotid, tongue, oral cavity, soft/hard palate, lip, salivary gland, gingiva, intra-oral mandible, and tonsil according to WHO/IARC tumor classification

^c^Nasal cavity/paranasal sinuses cancers include maxilla, maxillary sinus, sinonasal, nasal, and nasopharynx according to WHO/IARC tumor classification

^d^Hypopharynx/larynx/trachea cancers include larynx, Hypopharynx, glottis, vallecula, pharynx, neck (non-skin), and submandibular according to WHO/IARC tumor classification

^e^Defined as stages 3–4 and metastatic malignancies, % represents reported pathology department diagnosis

^f^Comorbidities include Comorbidities include malaria, pleural effusion, pneumonia, ascites, syphilis, liver cysts, oral thrush, oral ulcers, sinusitis, yellow fever, etc

Due to missing HIV status data in several medical records, a separate ORCI-based HIV care and treatment clinic (CTC) database was identified to supplement patient HIV status. This database is supported by the Tanzanian Ministry of Health and housed at ORCI. Linkage between patients contained in the NADC dataset and the HIV database was performed on-site by ORCI staff. This database provided additional HIV-positive data on 20 patients, 17 with previously missing data, 1 recorded as HIV-negative, and 2 previously recorded as HIV-positive.

### Statistical analysis

Trends in absolute numbers and proportions of NADCs compared to all cancers diagnosed at ORCI between 2002 and 2014 as well as 2010–2014 were analyzed using Joinpoint Regression Program, Version 4.2.0 - April 2015 [20, 21]. Categorical variables were examined using Chi square or Fisher’s exact tests and continuous variables were examined using t-test or Wilcoxon rank-sum test according to the normality of the data. Multinomial logistic regression models were used to model multiple nominal HIV status outcome variables for calculation of odds ratios and 95% confidence intervals to determine associations between patient HIV-status and covariates [22]. Data analysis was performed using SAS 6.4. The study was approved by the IRB committees of the University of Michigan and the Ocean Road Cancer Institute.

## Results

**Fig. 1:**
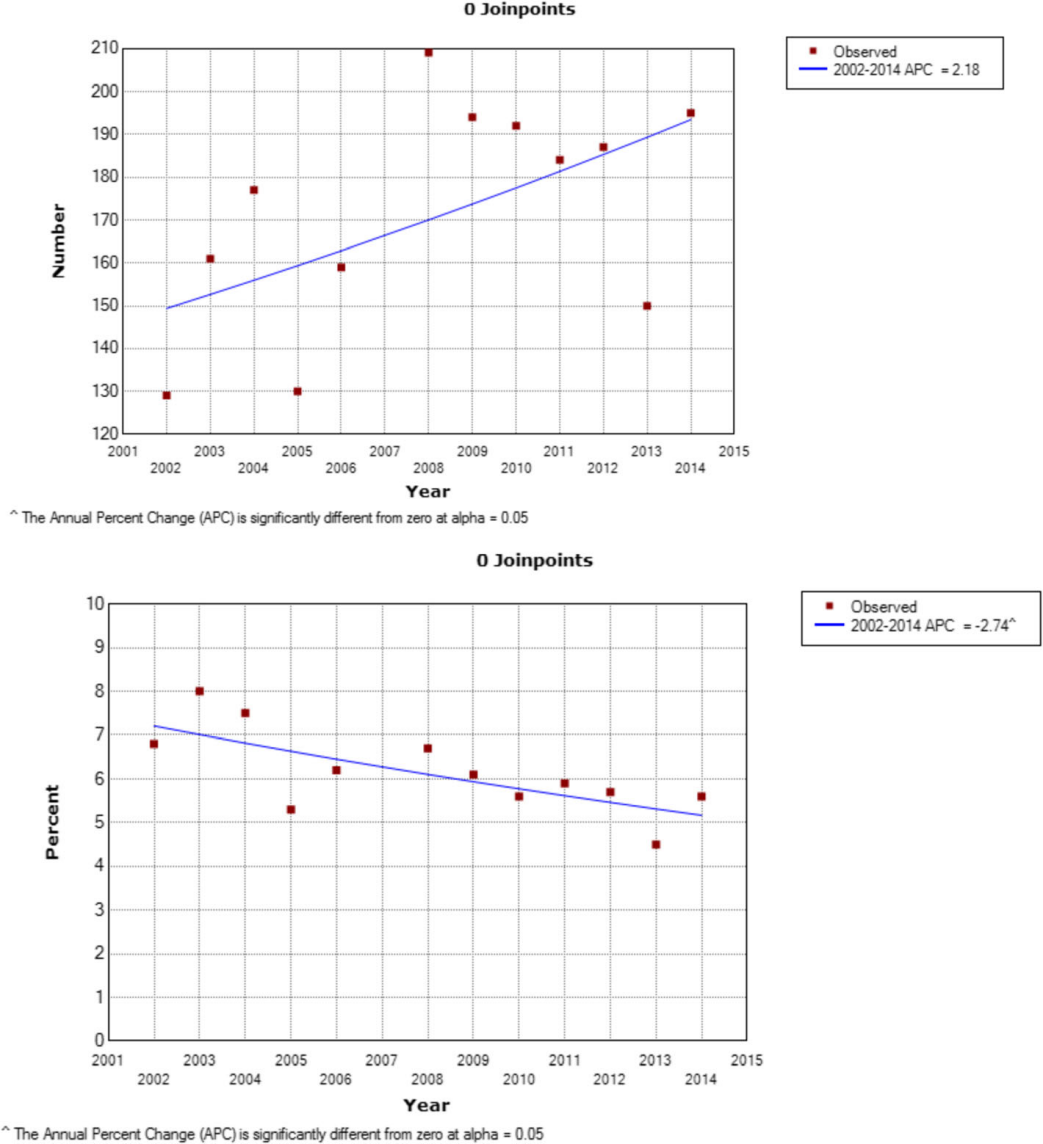
Number^a^ and proportion^b^ of lung, liver, and head and neck NADCs, 2002–2014. ^a^ Total number of lung, liver, and head and neck cancers diagnosed annually at ORCI according to logbooks. ^b^ Proportion of total lung, liver, and head and neck cancers diagnosed compared to all cancers diagnosed annually at ORCI according to logbooks

A total of 2067 patients with lung, liver, and head and neck cancers were recorded in the annual logbooks between 2002 and 2014 at the ORCI. Site specific numbers included 165 lung, 389 liver, and 1513 head and neck cancer patients presenting to ORCI according to hospital logbook records. Between 2002 and 2014, the absolute number of lung, liver, and head and neck NADCs diagnosed at ORCI increased by 33.8% (Joinpoint logarithmic annual percent change (APC) = 2.18% (Fig. 1). Site-specific increases were observed in the number of lung (APC = 7.67%), liver (APC 2002–2004 = 55.25%, APC 2004–2014 = 0.96%), and head and neck cancers (APC = 1.09%). The relative proportion of lung, liver, and head and neck NADCs compared to all cancers diagnosed at ORCI was 6.8% in 2002, exhibited a peak of 8.0% in 2003, and fell again to 5.6% in 2014 (2002–2014 APC = -2.74%) (Fig. 1). Relative proportions of lung and liver cancers increased (APC = 2.54%; 2002–2004 APC = 35.48%, 2004-2014 APC = -3.06% respectively), while the proportion of head and neck cancers significantly decreased compared to all cancers diagnosed at ORCI from 2002-2014 (APC = -3.78%).

Among the subset of pathologically confirmed cases from 2010 to 2014, for which we also abstracted information on HIV status, a total of 1127 patients were diagnosed with lung, liver, and head and neck cancers. We excluded five cases from our analyses, three who were less than 18 years of age and two who were pregnant at the time of diagnosis. When focusing only on this 5-year period, the absolute number of total NADC cases increased by 5.7% from 2010 to 2014 (APC = 4.23%). Specifically, numbers of pathologically confirmed lung (APC = 12.47%), liver (APC = 4.01%) and head and neck (APC = 3.34%) cancer cases all increased from 2010 to 2014. The relative proportion of pathologically confirmed NADCs compared to all cancer diagnoses increased from 5.8% in 2010 to 6.0% in 2014 (APC = 2.92%). Proportions of pathologically-confirmed lung (APC = 11.07%), liver (APC = 2.80%), and head and neck (APC = 2.07%) cancers each increased compared to total cancer diagnoses between 2010 and 2014.

HIV status was available for 270 (24%) of the 1127 NADC cases diagnosed during this 5-year period. The proportion of NADC patients that were recorded as HIV-positive decreased slightly from 8.1% in 2010 to 7.1% in 2014 (APC = -3.77%). Medical record abstraction alone identified 256 patients as having known HIV statuses (*n* = 63 HIV-positive; *n* = 193 HIV-negative) and 833 patients as having unknown HIV statuses. ORCI records provided 93.1% of known HIV statuses and 6.9% was identified through a separate HIV CTC clinic database. Linkage with this separate HIV database increased the number of patients with known HIV statuses to 270 (*n* = 81 HIV-positive; *n* = 189 HIV-negative) resulting in an HIV-positive status correction factor of 6.2% in NADC patients diagnosed between 2010 and 2014.

Table 1 shows demographic characteristics, HIV, and health status of lung, liver, and head and neck cancer patients. The distribution of gender between lung and liver cancers was 50.5 and 41.7% female, for the 2 cancers respectively. Nearly twice the percent of males were diagnosed with head and neck cancers compared to females (33.8% female, 66.2% male). Of all NADC cases, 11.7% of females were recorded to be HIV-positive, while only 4.6% of all males were recorded as HIV-positive, representing a female to male ratio of 2.5 in this NADC population.

Patients with cancer of the hypopharynx, larynx, and trachea regions of the head and neck had the oldest mean age at diagnosis (59.3 ± 13.4 years), while liver cancer patients had the youngest mean age at diagnosis (47.1 ± 15.6 years). Head and neck cancers, specifically those of the oral cavity and oropharynx as well as of the hypopharynx, larynx and trachea regions, had the most patients who had previously smoked (40.4% and 54.5% respectively) and previously consumed alcohol (43.2% and 48.2% respectively), while the majority of patients diagnosed with all other NADCs in this study indicated never smoking or drinking alcohol. The highest percentages of patients with recorded hepatitis B, hepatitis C, and cirrhosis diagnoses were in liver cancer patients (53.5%, 9.8%, and 71.1% respectively). Lung cancer patients had the greatest percentage of tuberculosis diagnoses (25.7%), the largest mean number of comorbidities (0.97 ± 0.98), and the largest percentage of patients with at least one subsequent cancer diagnosis (33.7%) compared to liver and head and neck cancer patients. The largest proportion of recurrence was recorded in 4.7% of all head and neck cancers, and specifically 7.8% of patients diagnosed with cancer of the nasal cavity or paranasal sinuses, while no recorded cases of recurrence were found in lung or liver cancer patients. The largest proportion of recorded deaths, 21.9%, occurred in individuals diagnosed with liver cancer compared to patients with lung cancer and all as well as sub-categories of head and neck cancers.

Of NADC patients, 4.0% of lung, 7.3% of liver, and 7.5% of all head and neck cancer cases were HIV-positive. Of patients known to be HIV-positive, 4.9% were diagnosed with lung cancer, 13.6% had liver cancer, and 81.5% were diagnosed with a head and neck cancer. Specifically, 44.4% of HIV-positive patients were diagnosed with cancer of the oral cavity/oropharynx, 27.2% had cancer of the nasal cavity/paranasal sinuses, and 9.9% had cancer of the hypopharynx/larynx/trachea (see footnotes of Table 1). When stratified by year, a nearly 25% increase in HIV-positive NADC cases occurred between 2012 and 2013, with HIV-positive head and neck NADC patients representing the majority of this increase. During the 5-year period from 2010 to 2014, HIV-positive NADC patients were diagnosed at a younger average age, decreasing from 49.8 years in 2010 to 44.1 years of age in 2014.

Table 2 shows NADC patient characteristics and compares differences in these variables according to HIV-status, either –positive, -negative, or unknown. P-values and odds ratios represent the comparison of HIV-positive patients and individuals with an unknown HIV status to those recorded to be HIV-negative. Compared to HIV-negative NADC patients, both HIV-positive and HIV- unknown patients were more likely to be female, with HIV-positive patients having more than twice the odds of HIV-negative patients (OR = 2.78; 95% CI 1.63, 4.74). Both HIV-positive and HIV-unknown individuals were more likely to reside in Dar es Salaam, have undergone treatment with radiotherapy, and have reported ever using alcohol compared to HIV-negative individuals, while HIV-positive patients were significantly less likely than HIV-negative patients to have reported ever smoking. Both HIV-positive and –unknown NADC patients were likely to have been diagnosed with fewer comorbidities, and were less likely to experience malignant recurrence compared to HIV-negative patients (OR = 0.54; 95% CI 0.29, 1.00). HIV-positive patients were significantly more likely to have been diagnosed with tuberculosis (OR = 2.31; 95% CI 1.05, 2.12) and were more likely to have recorded mortality while HIV-unknown patients were less likely to exhibit these characteristics compared to HIV-negative patients. Individuals having an unknown HIV status were significantly less likely to be diagnosed with an advanced stage malignancy (OR = 0.26; 95% CI 0.06, 1.08) as well as a subsequent cancer diagnosis (OR = 0.64; 95% CI 0.42, 0.97) and less likely to have undergone chemotherapy when compared to HIV-negative NADC patients.

**Table 2:** Characteristics of lung, liver, and head and neck NADC patients by HIV status, 2010-2014^a^

	HIV-negative (*N* = 189) N (%)	HIV-positive (*N* = 81) N (%)	*P* value^b^ HIV-positive vs. -negative	OR (95% CI)^c^ HIV-positive vs. -negative	HIV-unknown (*N* = 857) N (%)	*P* value^b^ HIV-unknown vs.-negative	OR (95% CI)^c^ HIV-unknown vs. -negative
Female	65 (34.4%)	48 (59.3%)	0.0001*	2.78 (1.63, 4.74)	297 (34.7%)	0.9449	1.01 (0.73, 1.41)
Mean Age ± SD	48.1 ± 15.6	47.6 ± 12.6	0.7878	1.00 (0.98, 1.02)	56.3 ± 15.7	<0.0001*	1.03 (1.02, 1.05)
Residence in Dar es Salaam	72 (38.1%)	41 (50.6%)	0.0560	1.67 (0.99, 2.82)	335 (39.1%)	0.7768	1.05 (0.76, 1.45)
Advanced cancer stage^d^	115 (60.8%)	42 (51.9%)	n/a	n/a	498 (58.1%)	0.0447*	0.26 (0.06, 1.08)
Cancer Treatment Modality							
Radiotherapy	111 (58.7%)	56 (69.1%)	0.1221	1.59 (0.88, 2.85)	603 (70.4%)	0.0023*	1.70 (1.21, 2.39)
Chemotherapy	97 (51.3%)	44 (54.3%)	0.6498	1.13 (0.70, 1.95)	433 (50.5%)	0.7092	0.94 (0.68, 1.30)
Tuberculosis	20 (10.6%)	19 (23.5%)	0.0364*	2.31 (1.05, 2.12)	55 (6.4%)	0.2532	0.71 (0.39, 1.28)
Co-morbidities^e^							
Mean count ± SD	0.59 ± 0.80	0.41 ± 0.75	0.0679	0.74 (0.52, 1.05)	0.44 ± 0.74	0.0720	0.80 (0.65, 0.95)
Smoking Status							
Ever use	71 (37.6%)	19 (23.5%)	0.0492*	0.54 (0.29, 1.00)	339 (39.6%)	0.4543	1.14 (0.81, 1.62)
Never	84 (44.4%)	42 (51.9%)	-	-	351 (41.0%)	-	-
Alcohol use							
Ever use	80 (42.3%)	29 (35.8%)	0.8749	1.07 (0.58, 2.00)	356 (41.5%)	0.6032	1.10 (0.77, 1.56)
Never	74 (39.2%)	25 (30.9%)	-	-	300 (35.0%)	-	-
Recurrence	9 (4.76%)	1 (1.2%)	0.2904	0.25 (0.03, 2.01)	31 (3.6%)	0.4577	0.75 (0.35, 1.60)
Subsequent cancer diagnoses	35 (18.5%)	15 (18.5%)	1.0000	1.00 (0.51, 1.95)	109 (12.7%)	0.0362*	0.64 (0.42, 0.97)
Recorded Death	28 (14.8%)	17 (21.0%)	0.2123	1.53 (0.78, 2.98)	104 (12.14%)	0.3153	0.79 (0.51, 1.25)

^a^Multinomial logistic regression comparing HIV-positive and HIV-unknown to HIV-negative patient group

^b^Chi-squared tests/Fisher’s exact test for categorical variables; t-tests/Wilcoxin rank-sum tests for continuous variables

^c^Odds Ratios (95% Confidence Intervals) calculated using multinomial logistic regression models (Reference = HIV-negative)

^d^Defined as stages 3–4 and metastatic malignancies, % represents reported pathology department diagnosis

^e^Comorbidities include malaria, pleural effusion, pneumonia, ascites, syphilis, liver cysts, oral thrush, oral ulcers, sinusitis, yellow fever, etc

*Significant at alpha <0.05

## Discussion

This study characterized the burden and proportional changes in distributions of lung, liver, and head and neck NADCs prior to and following ART introduction in Tanzania and revealed multiple notable findings. First, the number of lung, liver, and head and neck cancer diagnoses at ORCI increased by 33.8% from 2002-2014. Second, compared to all cancers diagnosed at ORCI from 2010-2014, we observed that proportions of lung and liver cancers increased, while the proportion of head and neck cancers significantly decreased compared to all cancers. Third, the number of pathologically confirmed lung, liver, and head and neck cancers increased from 2010-2014, although we observed a slight decrease in the proportion that were HIV-positive. Fourth, the largest proportion of HIV-positive cases were patients diagnosed with cancers of the head and neck. Lastly, over half the patients diagnosed with these NADCs during 2010-2014 presented to ORCI at advanced stages of malignancy.

Increasing numbers of lung, liver, head and neck cancers were observed from 2002-2014, following national ART implementation in 2004, but a proportional decrease was observed compared to all cancers diagnosed at ORCI. These findings should be placed within the context of a 45.8% increase of total cancers diagnosed at ORCI and a 30% increase in the Tanzanian population during the same time period [23]. Cervical, prostate, breast, Kaposi sarcoma, and esophageal cancers are among the most common types of malignancies in Tanzania [24]. While cervical, prostate, and breast cancers account for large proportions of the cancer burden in this population, the proportion of Kaposi sarcoma, an AIDS-defining malignancy, to all other cancers diagnosed is decreasing [24, 25]. Although NADCs evaluated in this study decreased in percentage over the 12-year period, ongoing evolution of risk factors and population characteristics in Tanzania may lead to increasing proportions of these types of malignancies in the future.

The increasing relative proportion of all pathologically confirmed lung, liver, and head and neck NADCs (APC = 2.92%) between 2010 and 2014 compares to higher relative increasing proportions of ano-rectal cancer, squamous cell carcinoma of the eye, and Hodgkin lymphoma NADC diagnoses among all cancers at ORCI from 2002-2012 reported in our recent study (APC = 4.68%) [11]. Potential reasons for noting a smaller magnitude of increase in this study include the smaller 5-year period of analysis, the short survival of lung and liver cancers that may have limited the inclusion of those cases from the ORCI site, limited sensitivity of some diagnostic tools [26–28], and lacking diagnostic imaging tools that are becoming more prevalent in developing countries for diagnosis of lung and liver cancers [29]. Similarly, head and neck cancers, especially those of the oral cavity, are diagnosed upon presentation of advanced visible symptoms rather than through early detection [30]. Larger numbers of head and neck cancers diagnosed in 2010 may represent accumulated undiagnosed burden while more current numbers may reflect annual rates, contributing to this observed proportional decrease compared to all ORCI cancer diagnosis. Further, risk factors contributing to head and neck cancer etiology such as tobacco and alcohol use, HPV, and other infections were largely unrecorded in the ORCI medical records and may influence the proportional decrease in cases noted when head and neck cancers were compared to all cancer diagnoses from 2010-2014 [31]. Ano-rectal cancer, squamous cell carcinoma of the eye, and Hodgkin lymphoma, as NADCs, are diagnosed earlier in pathogenesis, which makes underreporting of these cancers unlikely compared to the 3 groups of cancers examined in this study [32–34].

Comparison between NADC patient HIV status groups revealed expected differences between HIV-positive and -negative individuals, but also exhibited differences between the group of patients with unknown HIV status from those who were HIV-negative. This unknown-status group accounted for a large proportion of the sample and differed from the other 2 groups based on multiple factors. Significantly different characteristics of HIV-unknown versus HIV-negative patients include that HIV-unknown patients were likely to be older, less likely to be diagnosed at an advanced stage of malignancy, and less likely to have been diagnosed with a subsequent type of cancer after their initial diagnosis. Additionally, HIV-unknown NADC patients had fewer comorbidities, were less likely to have tuberculosis, and less likely to have recorded mortality suggesting that this patient population may be healthier than the HIV-negative and -positive NADC patient groups. Overall better health status in HIV-unknown NADC patients compared to those testing HIV-negative, based on the variables examined, may partially explain their lack of documented HIV sero-status, as less health care service utilization in the absence of other illnesses may contribute to less frequent referral to HIV testing. However, other barriers to HIV testing may exist for these patients and HIV-unknown patients cannot be assumed to be HIV-negative. In fact, the HIV-unknown patient group exhibited some similarities to the HIV-positive patient group including containing more females and being likely to have been treated with radiotherapy compared to HIV-negative patients.

Although only about one quarter of NADC patient HIV status was known, a female to male ratio of 2.5 was observed for HIV-positivity. This relationship exceeds the known ratio of HIV-positive females to males of 1.5 in the general Tanzanian population [35]. When NADC patients were compared based on HIV-status, HIV-positive individuals were more than twice as likely to be females compared to HIV-negative individuals, which adheres to, yet also exceeds, the disproportional national burden of HIV infection in women [35]. The proportion of HIV-positive patients diagnosed with lung, liver, and head and neck cancers decreased from 8.1 in 2010 to 7.1% in 2014, with a logarithmic annual change of -3.77%. This contrasts increasing proportions of HIV-positive NADC patients found in our recent study during 2002-2012 at ORCI (APC = 16.02%) as well as findings from a study conducted in neighboring Kampala, Uganda which revealed increasing incidence of lung, liver, and nasopharyngeal cancer in HIV-infected individuals between 1988-2002 [11, 36]. While decreasing prevalence of HIV-positivity was noted in this patient population over time, prevalence remained above 6% throughout 2010-2014 which consistently exceeds the 2013 national prevalence of 5% in Tanzania [9, 10]. Elevated prevalence of HIV-positivity within this NADC population may suggest HIV-related immunodeficiency as a contributing risk factor for developing NADCs, though more complete data will be necessary to confirm this association. The observed decrease in HIV-positive NADC patients in this study may be due to a large proportion of patients having an unknown HIV status. Missing HIV status data in ORCI files may be due to patients’ unwillingness to disclose their status to ORCI clinicians, incomplete record transfer from referring medical facilities, or patients not being recommended for or failing to complete testing. The slight reduction in HIV-positive NADC cases may further be influenced by improved longevity and decreased potential for HIV transmission of those managing infection with ART.

Head and neck cancer cases accounted for the largest proportion of HIV-positivity in this study. HPV infection, especially by HPV-16, has been commonly identified as a risk factor contributing to the onset of head and neck cancers, predominantly those of the oropharynx, tonsils, and the base of the tongue [31]. A recent cohort study found that the relative risk of carrying HPV decreased proportionately by 11% for each 100 CD4 cells/μL increase, suggesting plausible co-infection of HPV in immunosuppressed including HIV-positive individuals, as well as attenuating risk of HPV infection with improved immune function [37]. The large number of HIV-positive head and neck cancer cases in this study may be due to this co-infection, though more complete HPV status data in ORCI medical records will be necessary to confirm this association.

Over half of the lung, liver, and head and neck NADC patients diagnosed between 2010-2014 were identified as having stage III, IV, or metastatic malignancies. When separated by HIV status, no HIV-positive NADC patients had less than an advanced stage of cancer (stages III-IV or metastatic malignancy) while only two HIV-negative patients had malignancies diagnosed at a stage earlier than stage III. The ORCI and cancer care facilities at Bugundo Medical Centre in Tanzania are estimated to have the capacity to only serve about 5000 of the estimated 35,000 new cancer cases in Tanzania each year [24]. Limited cancer treatment resources and geographical barriers to seeking care delays presentation for disease management and contributes to the 80% mortality in Tanzanian cancer patients each year [24]. The majority of advanced stage NADCs received by ORCI reveals how late in pathogenesis patients present for treatment and the lack of early-stage cancers contained in the ORCI hospital-based cancer registry. Due to the urgency of initiating treatment in these cases, ORCI medical records do not always contain patient history details such as past and current comorbidities or substance use. The tendency for this data to be contained in medical records from referring hospitals and clinics identifies these entities as the chief source of data on patients’ lifetime health.

In this study, the distribution of gender between lung and liver NADCs was nearly equal which is consistent with population data reported by East African Countries in *Cancer Incidence in Five Continents* [38]. Nearly twice the number of males were diagnosed with head and neck cancers compared to females, consistent with the male to female ratio of 2:1 previously found in head and neck cancer cases at Muhimbili National Hospital, Tanzania from 2012-2013 [39]. This study also observed the greatest percentage of tuberculosis diagnoses in lung cancer patients and hepatitis B, hepatitis C, and cirrhosis diagnoses in liver cancer patients. These findings within Tanzanian NADC population are consistent with previously documented risk factors contributing to lung and liver cancers [40, 41].

In contrast to the reported increasing average age of HIV-positive patients with ano-rectal cancer, squamous cell carcinoma of the eye, and Hodgkin lymphoma from 2002-2012 [11], we observed a decreasing average age in HIV-positive lung, liver, and head and neck NADC patients from 2010-2014. This difference may be due to the types of cancers that HIV-positive individuals were diagnosed with in this study compared to those included in our previous publication [11] and associated average ages of onset of these malignancies. In this study, HIV-positive patients diagnosed with cancer in 2010 were individuals having lung and head and neck cancers; no liver cancer cases were recorded as HIV-positive during this year. HIV-positive patients diagnosed with cancer in 2014 had liver and head and neck cancers; no lung cancer cases were recorded as HIV-positive during this year. Lung cancer usually occurs in those with more advanced age (≥65 years), while liver cancer tends to occur at a younger age (mean 34.7 years) in southern Africa, which may partially explain the observed decrease in HIV-positive patients’ average age during these 5 years [42, 43].

Epidemiologic research focused on non-AIDS-defining cancers in Tanzania is limited. This study of NADCs in patients presenting to ORCI has multiple strengths. This study builds upon work previously done in this population to analyze changing patterns of Kaposi’s sarcoma as well as work on three other NADCs in order to more broadly characterize changing cancer burden in Tanzania and contribute to knowledge for future research in this population [11, 25]. This large dataset of patients with lung, liver, and head and neck cancers contains cases that were pathologically confirmed and included individuals from a majority of Tanzania’s geographic regions. In the absence of a population-based cancer registry, the consistency of patient demographics by cancer site with those reported by *Cancer Incidence in Five Continents* suggests general comprehensiveness of the ORCI hospital-based cancer registry.

Consistent with methodology detailed in our previous study [11], this study was a secondary retrospective analysis of ORCI medical record data, for which abstraction was limited to the completeness of logbooks and patient files. As logbooks contained medical record numbers associated with cancer diagnoses, identification of medical records corresponding to these malignancies of interest was dependent on the quality of documentation by ORCI staff. Limitations related to abstraction of medical record data included patient history and appointment notes taken by multiple clinicians in differing formats and levels of detail. Due to the lack of routine HIV-screening at ORCI, many patients had missing HIV-statuses even when files indicated that patients had been tested. For this reason, additional HIV CTC clinic data was utilized and linked to this dataset. After HIV database linkage, 24.0% of lung, liver, and head and neck NADC patients had a known HIV-status which compares to the 30.0% of ano-rectal, squamous cell carcinoma of the eye, and Hodgkin lymphoma NADC patients with a supplemented HIV-status found in the previous NADC study at ORCI [11]. Due to the overall lack of consistent HIV status data in ORCI medical records, the slight decrease in HIV-positive NADC cases in this study should be interpreted with caution. Screening for additional cancer risk factors such as HPV is also not routinely completed at ORCI. Given the association between HPV infection and onset of head and neck cancers, more complete data would be informative in this context [31]. Limited available data on HIV and HPV infection in this hospital-based cancer population prevents a clear understanding of the driving force behind incidence of NADCs in Tanzania at this time.

Future research into NADCs in the Tanzanian population should include linkage of ORCI patient data with HIV CTC clinic data in order to verify and supplement HIV statuses of cancer patients. Since CTC clinic files are housed at ORCI, future studies should further utilize these files to understand additional factors contributing to NADC incidence such as HIV treatment details, CD4 count, co-infections, height and weight for BMI calculations, and more thorough substance use data. Over half of the patients in this study presented at ORCI with advanced stages of malignancy. To better capture cancer patients at earlier stages of disease, we recommend that projects focus on collecting data at regional health facilities, private health clinics, or other disease screening facilities (such as HIV or HPV) rather than collecting data on patients whose diseases have progressed to the point of presenting at ORCI for management. Earlier capture of NADC cases at referring health facilities has the potential to more completely inform patient histories of infection and co-morbidities, including hepatitis and cirrhosis, through routine blood panels. More complete data on risk factors and comorbidities from these health facilities will contribute to a better understanding of the etiology and pathogenesis that lead to development of uncommon non-AIDS-defining malignancies. Future studies should examine infectious and other risk factors contributing to the growing prevalence of these NADCs and within the HIV-positive population of Tanzania. With an increased understanding of the role of infectious agents in pathogenesis, intervention and management strategies can more accurately target and address the documented increasing prevalence of non-AIDS-defining malignancies in Tanzania.

## Conclusions

In conclusion, this study offers further evidence for increasing trends in non-AIDS-defining cancer frequency at the ORCI. Specifically, burden of lung, liver, and head and neck cancers have increased between 2002-2014 which coincides with elevated trends in ano-rectal cancer, squamous cell carcinoma of the eye, and Hodgkin lymphoma—three additional NADCs—recently reported from 2002-2012 in this population [11]. As a wider profile of NADC diagnoses is found to be increasing, evidence of changing cancer burden following national ART implementation grows stronger. Future research must examine trends in AIDS-defining cancers throughout this time period as well as the etiology of these and other NADCs as a contributor to increasing numbers of cancer diagnoses in Tanzania. Such research should examine disease status and duration of cancer patients infected by HIV, HPV, and hepatitis, as well as patient health status at earlier stages of cancer diagnosis documented at regional, private, and screening facilities. Additionally, this study further supports the necessary development of a National AIDS Control Program (NACP) database in Tanzania for the comprehensive and centralized recording of HIV/AIDS diagnoses, infection duration, and treatment in order to gain a more complete understanding of how HIV infection relates to trends in NADC prevalence.

## Abbreviations

ADC: AIDS-defining cancer
APC: Annual percent change
ART: Antiretroviral therapy
CTC: Care and treatment clinic
HIV: Human immunodeficiency virus
NADC: Non-AIDS-defining cancer
ORCI: Ocean Road Cancer Institute
